# Discontinuities in the endothelium of epiphyseal cartilage canals and relevance to joint disease in foals

**DOI:** 10.1111/joa.12391

**Published:** 2015-10-15

**Authors:** Ingunn Risnes Hellings, Stina Ekman, Kjell Hultenby, Nils Ivar Dolvik, Kristin Olstad

**Affiliations:** ^1^ Department of Companion Animal Clinical SciencesEquine SectionFaculty of Veterinary Medicine and BiosciencesNorwegian University of Life SciencesOsloNorway; ^2^ Department of Biomedical Sciences and Veterinary Public HealthSection of PathologySwedish University of Agricultural SciencesUppsalaSweden; ^3^ Department of Laboratory MedicineKarolinska Institutet and University HospitalHuddingeSweden

**Keywords:** cartilage canal, collagen type I, endothelium, epiphyseal growth cartilage, fenestrations, horses, osteochondrosis, ultrastructure

## Abstract

Cartilage canals have been shown to contain discontinuous blood vessels that enable circulating bacteria to bind to cartilage matrix, leading to vascular occlusion and associated pathological changes in pigs and chickens. It is also inconsistently reported that cartilage canals are surrounded by a cellular or acellular wall that may influence whether bacterial binding can occur. It is not known whether equine cartilage canals contain discontinuous endothelium or are surrounded by a wall. This study aimed to examine whether there were discontinuities in the endothelium of cartilage canal vessels, and whether canals had a cellular or acellular wall, in the epiphyseal growth cartilage of foals. Epiphyseal growth cartilage from the proximal third of the medial trochlear ridge of the distal femur from six healthy foals that were 1, 24, 35, 47, 118 and 122 days old and of different breeds and sexes was examined by light microscopy (LM), transmission electron microscopy (TEM) and immunohistochemistry. The majority of patent cartilage canals contained blood vessels that were lined by a thin layer of continuous endothelium. Fenestrations were found in two locations in one venule in a patent cartilage canal located deep in the growth cartilage and close to the ossification front in the 118‐day‐old foal. Chondrifying cartilage canals in all TEM‐examined foals contained degenerated endothelial cells that were detached from the basement membrane, resulting in gap formation. Thirty‐three percent of all canals were surrounded by a hypercellular rim that was interpreted as contribution of chondrocytes to growth cartilage. On LM, 69% of all cartilage canals were surrounded by a ring of matrix that stained intensely eosinophilic and consisted of collagen fibres on TEM that were confirmed to be collagen type I by immunohistochemistry. In summary, two types of discontinuity were observed in the endothelium of equine epiphyseal cartilage canal vessels: fenestrations were observed in a patent cartilage canal in the 118‐day‐old foal; and gaps were observed in chondrifying cartilage canals in all TEM‐examined foals. Canals were not surrounded by any cellular wall, but a large proportion was surrounded by an acellular wall consisting of collagen type I. Bacterial binding can therefore probably occur in horses by mechanisms that are similar to those previously demonstrated in pigs and chickens.

## Introduction

Cartilage canals are channels that carry blood vessels from the perichondrium into the specialised metaphyseal and epiphyseal growth cartilage of long bones during normal development (Blumer et al. 2008). The metaphyseal growth cartilage or growth plate (physis) is located between the primary ossification centre of the diaphysis and the secondary ossification centre of the epiphysis (Banks, 1993). The epiphyseal growth cartilage is located superficial to the secondary ossification centre and, together with the avascular articular cartilage, constitutes the articular–epiphyseal cartilage complex (Banks, 1993). The vascular component of epiphyseal growth cartilage canals consists of an afferent arteriole opening into glomerulus‐like capillaries that re‐join to form efferent venules that run in the same canal (Wilsman & Van Sickle, 1972; Hayashi, 1992). The vessels are embedded in loose connective tissue that contains undifferentiated mesenchymal cells (Lutfi, 1970a; Stockwell, 1971; Wilsman & Van Sickle, 1972). Cartilage canals have also been described to contain lymphatics (Wilsman & Van Sickle, 1972) and unmyelinated nerve fibres (Stockwell, 1971; Wilsman & Van Sickle, 1972; Hedberg et al. 1995). Some authors report that canals are surrounded by a cellular (Lutfi, 1970a; Doménech‐Ratto et al. 1999) and/or an acellular wall (Lutfi, 1970a; Stockwell, 1971; Haines, 1974; Doménech‐Ratto et al. 1999). The cartilage canals are considered important for supplying the growth cartilage with nutrients and removing waste (Lutfi, 1970a; Wilsman & Van Sickle, 1972). Undifferentiated perivascular mesenchymal cells may contribute to interstitial growth of the cartilage model (Lutfi, 1970a; Wilsman & Van Sickle, 1972; Haines, 1974). Vascularised cartilage canals also appear to be critical for establishment of the secondary ossification centre, and perivascular mesenchymal cells may act as a source of osteoblasts for bone formation (Kugler et al. 1979; Blumer et al. 2005, 2007).

As the animal matures, the layer of epiphyseal growth cartilage becomes progressively thinner and the blood supply is gradually lost. The loss occurs through two distinct processes; chondrification and incorporation into bone (Haines, 1974; Carlson et al. 1991, 1995; Ytrehus et al. 2004b; Olstad et al. 2008b). Chondrification means that the canal becomes filled with cartilage; this process has not been associated with disease and is therefore considered physiological (Ytrehus et al. 2004a). Incorporation of vessels into bone has, however, been associated with the development of osteochondrosis (OC) in piglets and foals (Ytrehus et al. 2004b; Olstad et al. 2008b). The morphology of early lesions at predilection sites indicated that cartilage canal vessels failed at the point where they were incorporated into bone (Ytrehus et al. 2004b; Olstad et al. 2008b), leading to ischaemic chondronecrosis and the focal delay in endochondral ossification that is characteristic of OC (Ytrehus et al. 2007). The cartilage superficial to an OC lesion can fracture (Olstad et al. 2013), leading to mineralised fragments within the joint known as osteochondrosis dissecans (OCD; Ytrehus et al. 2007). A heritable predisposition for OC has been documented in horses (Grøndahl & Dolvik, 1993) and pigs (Reiland et al. 1978).

The blood supply to growth cartilage has also been implicated in acquired orthopaedic infections in children, piglets and chicks (Trueta, 1959; Speers & Nade, 1985; Denecke & Trautwein, 1986; Denecke et al. 1986). Bacteria were injected into the joints of piglets (Denecke & Trautwein, 1986; Denecke et al. 1986) and circulation of chicks (Emslie & Nade, 1983; Speers & Nade, 1985; Alderson et al. 1986). Within 12 h, the bacteria were bound to the extracellular matrix (ECM) of the growth cartilage (Emslie & Nade, 1983; Speers & Nade, 1985; Denecke & Trautwein, 1986; Denecke et al. 1986). Cartilage canals were previously observed to contain fenestrated vessels (Hunt et al. 1979; Howlett, 1980), and vascular discontinuities brought the contents of the circulation into direct contact with the cartilage (Speers & Nade, 1985; Alderson et al. 1986). The bacterial cell wall or glycocalyx appeared to express an affinity for the cartilage (Speers & Nade, 1985; Alderson et al. 1986), and surface proteins with specific binding affinity for different ECM components have since been demonstrated in relevant bacterial species (Chagnot et al. 2012). Within 24–96 h, the vessels where bacterial binding occurred were occluded, either by bacteria (Emslie & Nade, 1983; Alderson et al. 1986) or thrombi (Denecke & Trautwein, 1986; Denecke et al. 1986), and no longer provided a functional blood supply.

Detection of fenestrations requires transmission electron microscopy (TEM; Hunt et al. 1979; Howlett, 1980) and, as equine cartilage canals have so far only been examined by light microscopy (LM), it is currently unknown whether they contain fenestrated vessels. It is important to discover whether equine cartilage canals contain fenestrated vessels because, if bacterial binding can occur, it leads to occlusion of vessels (Emslie & Nade, 1983; Alderson et al. 1986; Denecke & Trautwein, 1986; Denecke et al. 1986), and vascular failure has been demonstrated to lead to OC and OCD (Olstad et al. 2013). Bacterial binding can therefore theoretically lead to fragments in joints by a similar mechanism, but for a different reason than OC. This is supported by clinical reports where cases with septic arthritis had OCD‐like flaps and fragments in the joints (Hance et al. 1993; Haggett et al. 2012). The prevalence of radiographic fragments was also higher in horses that survived infection before 6 months old than in comparable controls (Hendrickson et al. [Link]). Clearly, fragments that arise due to acquired infection must be managed differently from fragments that arise due to heritable OC. If animals with acquired fragments are excluded from breeding, this may fail to reduce the prevalence of heritable OC.

The aim of the current study was to examine whether there were discontinuities in the endothelium of cartilage canal vessels, and whether canals had a cellular or acellular wall, in the epiphyseal growth cartilage of foals.

## Materials and methods

Material was collected from six foals presented for routine post mortem examination at the Norwegian University of Life Sciences during the breeding seasons of 2012–2014. Included foals were assigned ascending case numbers from 1 to 6 (Table 1). There was no minimum age, but an upper age limit of 6 months was imposed. Both sexes and any breed of horse or pony were included. Clinical history and reason for death were recorded (Table 1), and foals with a history of systemic or local orthopaedic infections were excluded. In previous studies, an upper limit of 3 days from death to sample collection was imposed without signs of compromise to cell morphology on LM. However, during collection for the present study, signs of endothelial autolysis were found on TEM examination of material from two foals that had been dead more than 10 h before sample collection. Material from foals that had been dead more than 2 h was therefore excluded from TEM evaluation of the endothelium (Table 1). An upper limit of 24 h from death to collection was imposed for the TEM evaluation of all other structures, and TEM was therefore not performed on foal 3 (Table 1). For reasons unrelated to the study aim, results of LM examination are not reported for foal 6 (Table 1).

**Table 1:** Age, sex, breed, history and material available from included foals

Foal number	Age	Sex	Breed	Clinical history and reason for death	Hind limb sampled	Time from death to sampling	LM	TEM	
								Endo‐thelium	Other structure
1	1 day	Male	Norwegian Fjord	Healthy, but killed as part of an approved research project	Right	1–2 h	x	x	x
2	24 days	Male	Norwegian Coldblooded Trotter	Congenital haemangio‐endothelioma left stifle	Right	1 h	x	x	x
3	35 days	Male	Thoroughbred	Acute entero‐colitis	Both	24–36 h	x	–a	–a
4	47 days	Female	Standardbred	Constipated, emaciated, heart murmur	Left	10–12 h	x	–	x
5	118 days	Male	Norwegian Fjord	*Parascaris equorum* impaction, urinary tract rupture	Left	< 1 h	x	x	x
6	122 days	Male	Standardbred	Colic, *Parascaris equorum* impaction	Both	10–12 h	–b	–	x

LM, light microscopy; TEM, transmission electron microscopy.

a Material from foals that had been dead more than 24 h was not examined with TEM.

b For reasons unrelated to the study aim, results of LM examination are not reported for foal 6.

### Collection protocol

**Figure 1:**
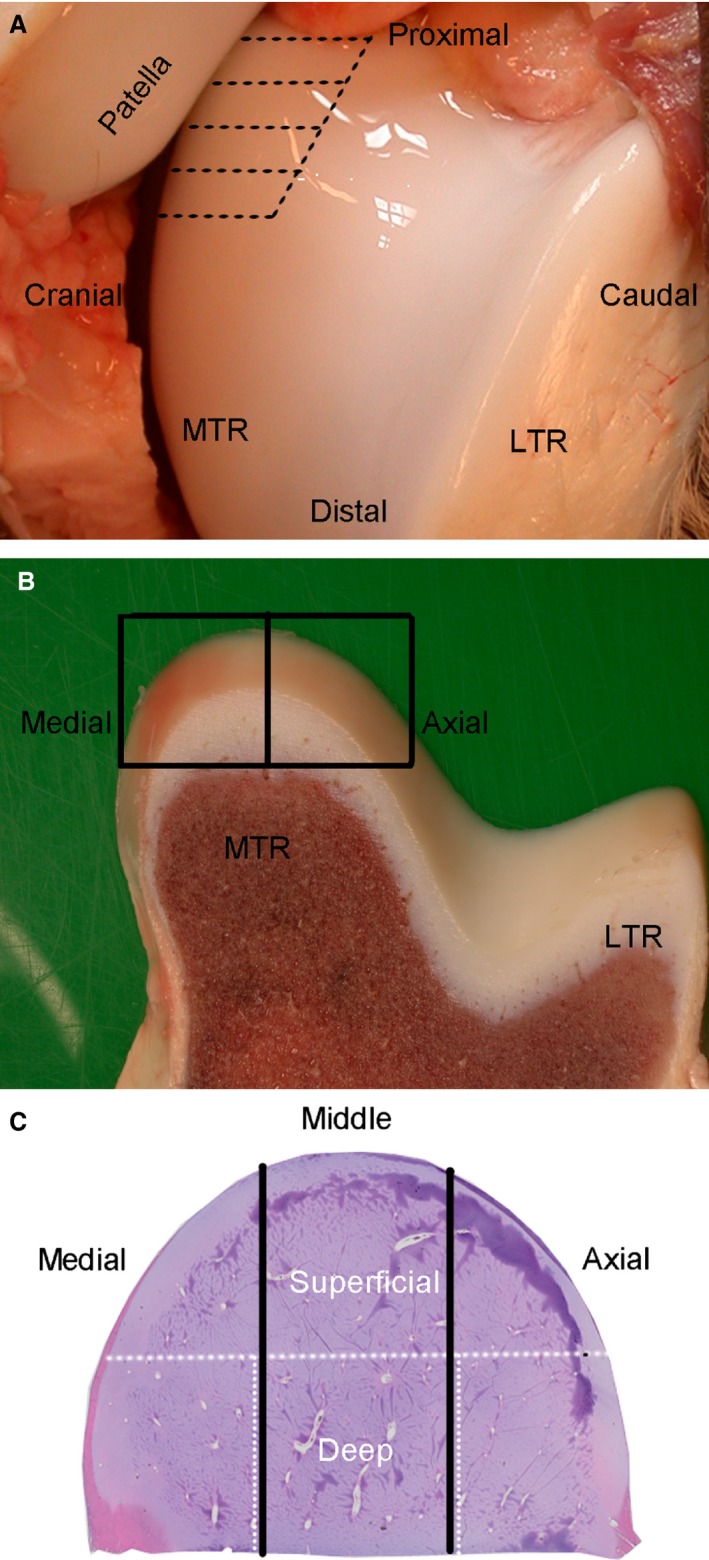
Method of collection. (A) Parallel cuts (stippled lines) were made in the proximal third of the medial trochlear ridge (MTR). LTR, lateral trochlear ridge. (B) Alternate adjacent either whole (both boxes) or half (single box) slabs were collected and fixed in formaldehyde for LM or paraformaldehyde/glutaraldehyde for TEM examination. (C) For TEM, areas containing cartilage canals were selected from the superficial and deep parts of the epiphyseal growth cartilage (white stippled line). For LM examination, the location of canals was recorded by dividing the MTR into medial, middle and axial thirds (black solid lines).

The skin on the cranial aspect of the distal femur was incised and the underlying soft tissues were reflected. The femoro‐patellar joint capsule was opened, and the lateral and medial trochlear ridges were exposed by dislocating the patella proximo‐medially (Fig. 1A). Parallel cuts, spaced 3–4 mm apart, were made in the cartilage covering the proximal third of the medial trochlear ridge, in an approximately horizontal plane that was parallel with the distal articular weight‐bearing surface (Fig. 1A). In foals 1, 2, 4 and 6, the cuts were made with a scalpel blade and the cut cartilage was separated from the femur by a second, approximately vertical cut that was made through the deepest portion of the cartilage, but superficial to the ossification front in order to avoid decalcification (Table 2). In foals 3 and 5, the cuts were made with a thin, hand‐held saw blade and the vertical cut was made through bone with the same blade, in order to include the ossification front (Table 2). Alternate adjacent either whole or half slabs (Fig. 1B; Table 2) were fixed in 4% phosphate‐buffered formaldehyde for LM, or 2% paraformaldehyde/2% glutaraldehyde in 0.1 m cacodylate buffer at pH 7.4 for TEM. After 48 h, slabs from foals 3 and 5 were decalcified in 10% ethylenediaminetetraacetic acid (EDTA). A minimum of two 5‐μm‐thick sections were cut from each formaldehyde‐fixed slab and stained with haematoxylin and eosin (HE) or toluidine blue (TB), respectively. The sections were examined in an Axio Lab.A1 microscope (Zeiss, Oberkochen, Germany) and photographed using an AxioCam ERc 5s (Zeiss).

**Table 2:**
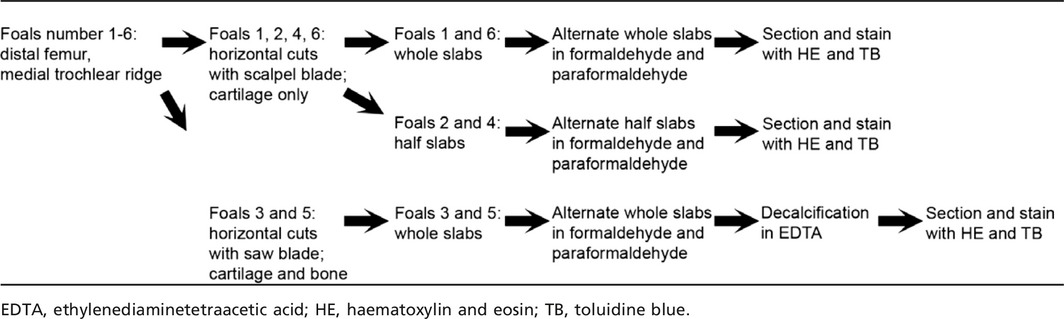
Summary of sample processing

Areas were selected from the superficial and deep parts (Fig. 1C) of the epiphyseal growth cartilage in the paraformaldehyde‐fixed slabs and trimmed down to 0.3 mm thickness × 1 mm latero‐medial width × 2 mm cranio‐caudal height. The trimmed samples were rinsed in 0.15 m cacodylate buffer and post‐fixed in 1% osmium tetroxide for 2 h at 4 °C, rinsed and dehydrated in ethanol, followed by acetone, and embedded in epoxy resin LX‐112 (Ladd, Burlington, VT, USA). Semi‐thin sections (0.5 μm) were cut and stained with TB and examined with LM in order to select areas for preparation of ultra‐thin sections (approximately 60–70 nm). The ultra‐thin sections were picked up on formvar‐coated copper grids, contrasted with uranyl acetate and lead citrate, and examined at 100 kV in a Tecnai 12 Spirit Bio TWIN TEM (FEI Company, Eindhoven, the Netherlands). Digital images were captured using a Veleta camera (Olympus Soft Imaging Solutions, GmbH, Münster, Germany).

### Immunohistochemical staining

Selected formalin‐fixed blocks were immunostained for collagen type I. A rabbit polyclonal anti‐collagen I antibody (ab34710; Abcam, Cambridge, UK) was used at a dilution of 1 : 100. The full staining protocol is available in Data S1.

### Parameters observed

The criteria used for LM evaluation were identical to those previously described in Carlson et al. (1991) and Olstad et al. (2007). Each cartilage canal was allocated an individual number to ensure that it was only registered once. The location of the canal was recorded according to the terminology illustrated in Fig. 1C. The ultrastructural features of the cartilage canals were described according to previous published criteria (Carlson et al. 1985, 1989; Ekman et al. 1990; Blumer et al. 2004a).

## Results

Material from six foals aged 1, 24, 35, 47, 118 and 122 days (Table 1) was studied. A total of 248 sectioned portions of cartilage canals were examined with LM. Selected portions of 43 cartilage canals from the six foals were examined using TEM.

### Discontinuities in the endothelium of patent cartilage canals

Two‐hundred‐and‐one of the 248 cartilage canals examined with LM were patent. Thirty patent canals were examined with TEM.

**Figure 2:**
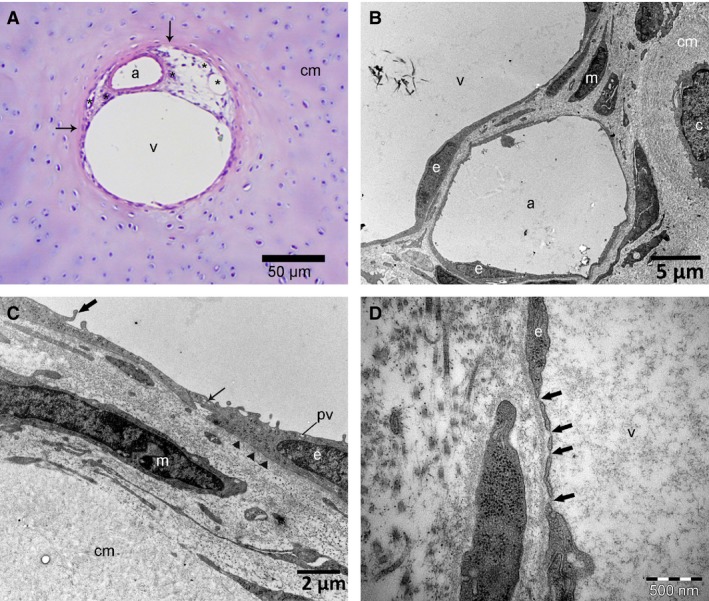
Patent canals on LM and TEM. Distal femoral epiphysis, medial trochlear ridge. (A) One‐day‐old, Norwegian Fjord. HE, 100 ×. Resting zone. The image shows a patent cartilage canal containing a thick‐walled arteriole (a), a thin‐walled venule (v) and multiple small capillaries (asterisks) lined by a continuous layer of endothelial cells with well‐defined nuclei. The vessels are embedded in loose connective tissue with spindle‐shaped mesenchymal cells. The cartilage canal is surrounded by a narrow zone of intensely eosinophilic‐staining matrix (arrows). This eosinophilic ring appears to fade into the surrounding basophilic hyaline cartilage matrix (cm). (B) TEM from the same foal as in (A), superficial canal. The image shows part of a small arteriole (a) and a venule (v) surrounded by spindle‐shaped mesenchymal cells (m) with flat long cytoplasmic processes and scant cytoplasm. Vascular structures are lined by one layer of continuous endothelium (e). A chondrocyte (c), surrounded by cartilage matrix (cm), is visible outside the cartilage canal. (C) Higher magnification of the same canal as in (B). Interdigitating tight junctions (thin arrow) are present between endothelial cells (e). The lamina densa of the basal membrane is visible as a thin, electron‐dense layer (arrowheads) beneath the endothelial cell. Endothelial cells contain free ribosomes together with pinocytotic vesicles (pv) and have finger‐like cytoplasmic projections (thick arrow), indicating active protein production and proliferation. cm, cartilage matrix; m, mesenchymal cell. (D) One‐hundred and eighteen‐day‐old Norwegian Fjord. TEM, deep canal, close to the ossification front. The image shows part of a venule (v) where the endothelial cell (e) cytoplasm is punctuated by fenestrations (arrows) that measure approximately 50 nm in length and has a thin, electron‐dense diaphragm.

On LM, patent canals contained vascular structures lined by normal endothelium (Fig. 2A). Vessels were embedded in a variable amount of loose connective tissue with perivascular spindle‐shaped mesenchymal cells. Canals were surrounded by chondrocytes and ECM similar to the zone that the canal was located in (Fig. 2A). Canals that were located centrally and deep in the cartilage tended to be large in diameter, whereas canals that were located close to the articular surface tended to be small. The vasculature in the large canals consisted of one thick‐walled arteriole, from one to 15 capillaries and one or more thin‐walled venules (Fig. 2A). Vessels in the largest canals were surrounded by layers of mesenchymal cells embedded in an ECM that did not stain with HE (Fig. 2A). The smallest canals contained only capillaries lined by thin endothelium that was in direct contact with the surrounding cartilage matrix. The endothelium of all patent canal vessels appeared intact on LM (Fig. 2A).

On TEM, vascular structures were lined by one layer of continuous endothelium that was supported by a basal membrane (Fig. 2B,C). The endothelial cell layer was extremely thin in places, and interdigitating tight junctions were present between adjacent cells (Fig. 2C). In the 1‐day‐old foal, endothelial cells displayed characteristics of active protein production and proliferation, such as dilated rough endoplasmic reticulum, plentiful free ribosomes and a rich presence of well‐preserved mitochondria together with numerous pinocytotic vesicles and finger‐like cytoplasmic processes, indicating viability and growth (Fig. 2B,C). In the 118‐day‐old foal, the endothelium of one venule located deep in the epiphyseal cartilage closer to the ossification front was punctuated by fenestrations that measured approximately 50 nm in length (Fig. 2D). The endothelial cells were normal, without signs of degeneration or regression. In the fenestrations, the contents of the circulation were separated from the surrounding cartilage matrix by a thin diaphragm (Fig. 2D). The fenestrations were present in two locations in the same venule, over a distance of 1 and 4 μm, respectively. The remaining endothelium was continuous, but extremely thin and in direct contact with the surrounding cartilage matrix. Fenestrations were not observed in the endothelium of the patent canals of the younger foals (1 and 24 days old). Lymphatics or nerves were not observed in any of the patent cartilage canals.

### Discontinuities in the endothelium of chondrifying cartilage canals

Forty‐seven chondrifying cartilage canals, comprising 24 early and 23 late chondrifying canals, were examined with LM. Thirteen chondrifying canals were examined with TEM.

**Figure 3:**
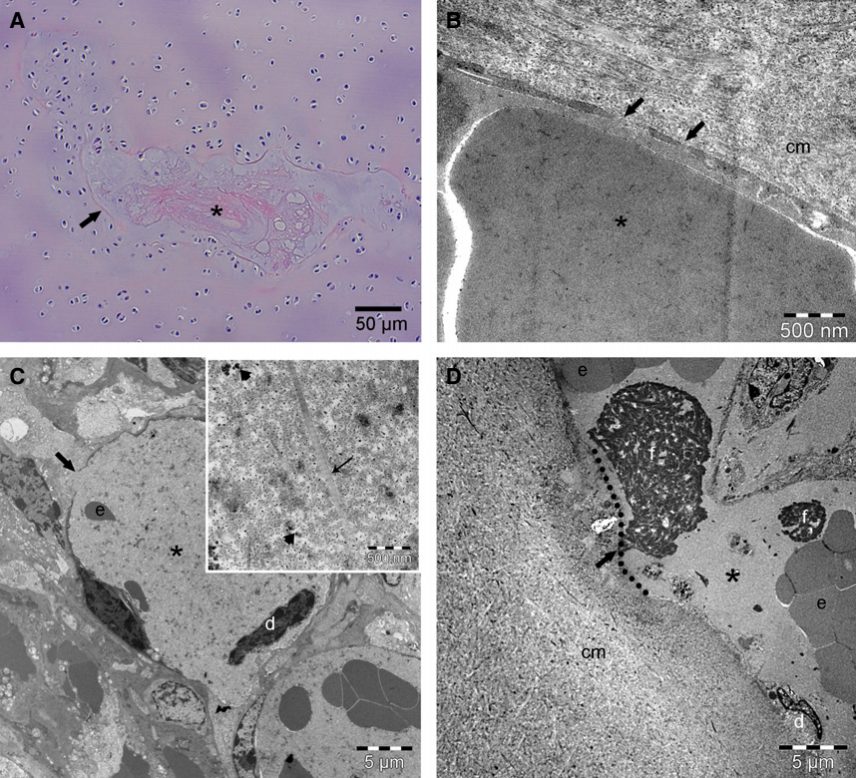
Chondrifying cartilage canals on LM and TEM. Distal femoral epiphysis, medial trochlear ridge. (A) Thirty‐five‐day‐old Thoroughbred. LM, HE, 100 ×. Proliferative zone. The endothelial lining of vessels is absent. The ghost remnant of a vascular lumen (asterisk) is filled with a mixture of eosinophilic, amorphous and basophilic, mucinoid material. The canal is surrounded by viable chondrocytes and a faint and interrupted eosinophilic ring (arrow). (B) Twenty‐four‐day‐old Norwegian Coldblooded Trotter. TEM from superficial canal, close to the articular surface. Variably‐sized, intermittent gaps (arrows) < 100 nm long are visible in the endothelial lining. The gaps have no visible diaphragm, and an erythrocyte (asterisk) inside the vessel lumen is in direct contact with the cartilage matrix (cm) surrounding the canal. (C) one‐hundred and twenty‐two‐day‐old Standardbred. TEM from deep canal. Detached endothelial cells (d) and a few erythrocytes (e) are visible in the vascular lumen (asterisk). There are gaps in the endothelial lining (thick arrow). Inset: higher magnification showing immature matrix inside the vascular lumen with single collagen fibrils (thin arrow) and proteoglycans (arrowheads). (D) One‐hundred and eighteen‐day‐old Norwegian Fjord. TEM from deep canal. A vessel (asterisk) in a chondrifying canal is lined by degenerate endothelial cells (d). There is a large gap (dotted line) in the endothelial lining. Thrombi, consisting of fibrin (f) and platelets are visible within the vascular lumen, one of which appears to be adhered (arrow) to the extra‐canalicular cartilage matrix (cm).

On LM, the endothelium of the chondrifying canals was either degenerate or absent (Fig. 3A). In early chondrifying canals, vascular structures were surrounded by a mixture of perivascular mesenchymal cells and some chondrocyte‐like cells. Late chondrifying canals were characterised by an absence of intact vessels and instead contained chondrocytes surrounded by intensely basophilic‐staining matrix. Ghost remnants of vascular lumina were occasionally present. The chondrifying canals were surrounded by viable chondrocytes (Fig. 3A).

On LM, chondrification was first observed in the distal terminus of the cartilage canal, close to the articular cartilage and towards the axial and medial aspects of the sample, and progressed proximally and centrally towards the ossification front with increasing age. In the 118‐ and 122‐day‐old foals, all cartilage canals close to the articular cartilage were in the process of chondrifying, or were chondrified. Corresponding portions of cartilage canals were therefore examined by TEM in the younger foals because they were considered representative of portions where the chondrification process was likely to start. Vessels inside these superficial canals were lined by an extremely thin endothelium. Cytoplasmic vacuolation, indicative of early degeneration, was present in some of the endothelial cells. Additional degenerative changes, including extensive cytoplasmic glycogen accumulation and separation of endothelial cells from the basal lamina, were more advanced in the 24‐day‐old (Fig. 3B) compared with the 1‐day‐old foal. These degenerative changes were compatible with regression of the endothelial cells as part of the physiological process of chondrification. The observed detachment of endothelial cells did, however, result in variably‐sized, but < 100‐nm‐long gaps intermittently along the endothelial lining. The gaps had no visible diaphragm, thus the contents of the circulation were in direct contact with the surrounding cartilage matrix at each gap (Fig. 3B). Similar changes were also present in the endothelium of portions of cartilage canals located immediately deep to overtly chondrifying canals in the 118‐ and 122‐day‐old foals (Fig. 3C). The gaps were larger in late, compared with early, chondrifying canals (Fig. 3C,D). In some cases, thrombi containing fibrin and platelets were seen adhered to the extra‐canalicular matrix via large gaps in the endothelium (Fig. 3D).

### Cellular wall

**Figure 4:**
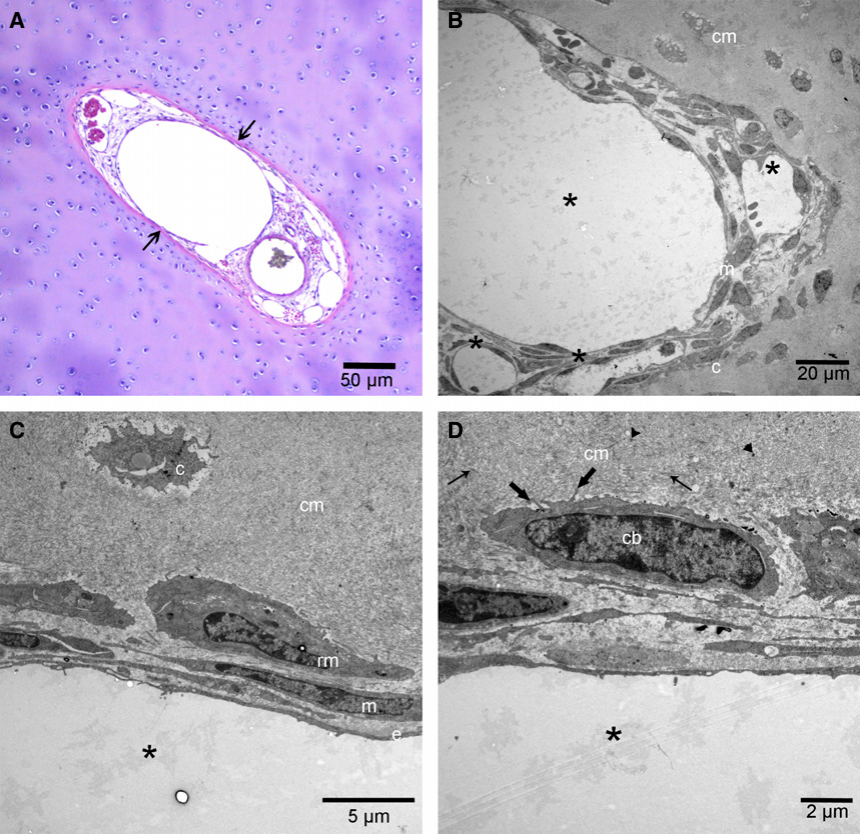
Cells surrounding cartilage canals on LM and TEM. Distal femoral epiphysis, medial trochlear ridge. (A) One‐day‐old, Norwegian Fjord. LM, HE, 100 ×. Patent cartilage canal, resting zone. The cartilage canal is surrounded by a zone of increased cell density compared with the density of chondrocytes between adjacent canals. The innermost layer of this hypercellular zone consists of spindle‐shaped cells that resemble mesenchymal cells, with sparse basophilic cytoplasm and no lacuna. The intermediate layer consists of chondrocyte‐like cells, surrounded by a lacuna and a slightly increased amount of amorphous basophilic ECM. The outermost layer consisted of single chondrocytes. The cartilage canal is also surrounded by a narrow zone of intensely eosinophilic‐staining matrix (arrows) sharply demarcated from the surrounding cartilage matrix. (B) Same animal as in (A). TEM from superficial canal, close to the articular surface. Multiple vessels (asterisks) are surrounded by mesenchymal cells (m). The canal is surrounded by chondrocytes (c) and cartilage matrix (cm). (C) Higher magnification TEM of (B). Fibroblast‐like mesenchymal cells (m) with long, flat cytoplasmic processes with scant cytoplasm are present innermost and closest to the endothelium (e) of vessels (asterisk). Rounded mesenchymal cells (rm) towards the periphery of the canal were interpreted as differentiation of mesenchymal cells towards a chondrogenic appearance. Outermost, a chondrocyte (c) in an electron‐lucent lacuna is surrounded by extra‐cellular matrix containing randomly oriented type II collagen fibrils and proteoglycans typical of mature hyaline cartilage matrix (cm). (D) Higher magnification TEM of (B). The image shows a chondroblast (cb) located on the border of a canal lumen between a vessel (asterisk) and the cartilage matrix (cm), situated in a narrow electron‐lucent lacuna and surrounded by a small amount of ECM containing proteoglycans (arrowheads) and collagen type II fibrils (thin arrows). This cell layer corresponded to the chondrocyte‐like cells seen in the intermediate layer on LM as shown in (A). Ruffled cytoplasmic processes (thick arrows) are present on the side facing the cartilage matrix.

Intra‐ and extra‐canalicular structures were not separated by any continuous cell lining or wall on LM or TEM in any of the examined cartilage canals. On LM, the majority of the canals were surrounded by chondrocytes that were similar to the zone that the canal was located in. However, 81/248 (33%) of all cartilage canals were surrounded by a narrow zone where cell density was increased relative to the density of chondrocytes between adjacent canals (Fig. 4A; Table 3). This hypercellular rim was most often found around cartilage canals in the youngest foal (Table 3).

**Table 3:** Number of cartilage canals surrounded by a hypercellular rim or an eosinophilic ring in light microscopic sections

Foal number	1	2	3	4	5	All 5 foals
Age	1 day	24 days	35 days	47 days	118 days	1–118 days
Whole or half slab	Whole	Half	Whole	Half	Whole	–
Hypercellular rim/total canals examined (%)	45/97 (46)	7/27 (26)	14/70 (20)	12/34 (35)	3/20 (15)	81/248 (33)
Eosinophilic ring/total canals examined (%)	67/97 (69)	19/27 (70)	48/70 (69)	24/34 (71)	12/20 (60)	170/248 (69)

On LM, the innermost layer of the hypercellular rim consisted of spindle‐shaped cells that resembled mesenchymal cells, with sparse basophilic cytoplasm and no lacuna (Fig. 4A). These cells were separated by a small amount of ECM. The intermediate layer consisted of cells that were chondrocyte‐like, with an increased amount of basophilic cytoplasm, and surrounded by a lacuna and a slightly increased amount of amorphous, basophilic ECM (Fig. 4A). The outermost layer consisted of single chondrocytes in lacunae that were separated by a further increased amount of hyaline cartilage ECM (Fig. 4A).

On TEM (Fig. 4B–D), the mesenchymal cells that were located centrally and close to the endothelium of vessels in canals with a hypercellular rim (Fig. 4B) appeared undifferentiated and fibroblast‐like (Fig. 4C). Cells towards the periphery of the canals were larger and rounder, representing mesenchymal cells differentiating towards a chondrogenic appearance (Fig. 4C). The ECM surrounding these cells was pale and electron‐lucent, contained little proteoglycan and occasional collagen type I fibrils (for collagen typing: see below), and was consistent with loose connective tissue (Fig. 4C). The intermediate layer was characterised by chondroblasts that were situated in electron‐lucent lacunae, surrounded by a small amount of ECM that contained proteoglycans and collagen type II fibrils (Fig. 4D, for collagen typing: see below). Outermost, chondrocytes were separated by a large amount of ECM that contained proteoglycans and the randomly oriented type II collagen fibrils that are typical of mature hyaline cartilage.

The above LM and TEM observations in canals surrounded by a hypercellular rim were interpreted as differentiation of perivascular mesenchymal cells into chondrocytes in order to contribute to growth of the epiphyseal cartilage model. The cartilage production observed on the margin of canals with a hypercellular rim resembled cartilage production during chondrification, but could be differentiated from it on the basis that hypercellular‐rimmed canals contained patent vessels.

### Acellular wall

On LM, some of the cartilage canals were surrounded by an ECM that was basophilic‐staining and similar to the inter‐territorial matrix between adjacent cartilage canals. Other cartilage canals were surrounded by a narrow zone of intensely eosinophilic‐staining matrix (Figs 2A, 3A and 4A). In TB sections, the eosinophilic ring corresponded to a zone of pale blue colouration, indicating low proteoglycan content. The eosinophilic ring was present in 170/248 (69%) of all cartilage canals (Table 3). Of the patent canals, 143/201 (71%) were surrounded by an eosinophilic ring. The proportion of canals surrounded by an eosinophilic ring in early and late chondrifying canals was 17/24 (71%) and 10/23 (44%), respectively. The eosinophilic ring was present around canals in both the proliferative and resting zones of the growth cartilage. The thickness of the eosinophilic ring varied around the circumference of any one canal. Sometimes the eosinophilic staining was sharply demarcated from the surrounding matrix, whereas other canals were surrounded by an interrupted and indistinct eosinophilic ring that faded into the surrounding basophilic hyaline cartilage matrix (Fig. 2A). When present around late chondrifying canals, the eosinophilic ring was always faint and interrupted (Fig. 3A).

**Figure 5:**
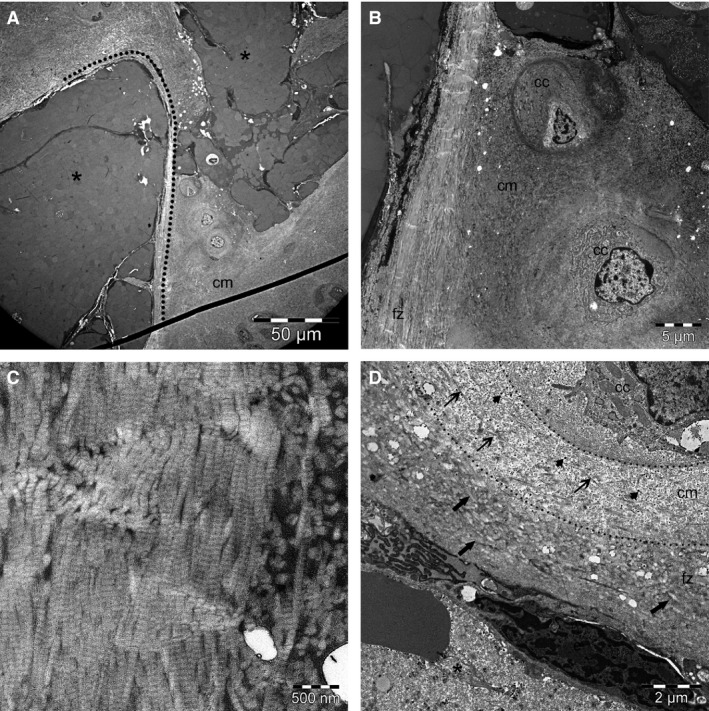
Cartilage canals surrounded by an acellular zone consisting of densely packed collagen type I collagen fibres on TEM. Distal femoral epiphysis, medial trochlear ridge. (A) One‐hundred and twenty‐two‐day‐old Standardbred. TEM, deep cartilage canal, close to the ossification front. Cartilage canal lumina (asterisks) are surrounded by a zone of densely packed, thick collagen type I fibrils and a relative absence of proteoglycans. The stippled line marks the transition between this acellular, fibrous zone and the cartilage matrix (cm). (B) Higher magnification TEM of the same canal as in (A). Collagen type I fibrils in the fibrous zone (fz) are densely packed and arranged parallel to each other, and differ from the appearance of the surrounding hyaline cartilage matrix (cm); cc, chondrocyte. (C) Higher magnification TEM of the same canal as in (B) showing the collagen type I fibril arrangement of the innermost zone surrounding the cartilage canal, characterised by densely packed, thick collagen fibrils up to approximately 90 nm diameter with a 64‐nm banding pattern. (D) TEM of same animal as in (B), superficial canal. The image shows the different zones of matrix surrounding a cartilage canal. The innermost fibrous zone (fz) contains thick, densely packed type I collagen fibres (thick arrows). Further out from the canal, the cartilage matrix (cm) contains randomly distributed individual collagen type II fibrils (thin arrows) embedded in a granular, proteoglycan‐rich (arrowheads) matrix. The margins of the outermost zone are marked by stippled lines and contain a mixture of type I and type II collagen. A chondrocyte (cc) is surrounded by a lacuna containing fine‐textured material. The degenerate endothelial lining of a chondrifying vessel (asterisk) is seen in the lower left corner of the picture. The vascular lumen contains single collagen fibrils, sparse proteoglycans and an erythrocyte.

A zone of matrix surrounding cartilage canals corresponding to the eosinophilic ring was examined with TEM (Fig. 5A–D). Proteoglycans and collagen were organised into distinct zones around some such canals. The innermost zone was characterised by thick, densely packed collagen fibres with a 64‐nm‐wide banding pattern interpreted as collagen type I, and a relative absence of proteoglycans (Fig. 5A–C). The individual collagen fibrils measured up to approximately 90 nm in diameter and were arranged parallel with each other (Fig. 5C). This zone measured from ≤ 1 μm up to 10 μm in thickness.

The intermediate zone contained randomly distributed individual collagen fibrils that were embedded in a granular proteoglycan‐rich matrix as is characteristic of hyaline cartilage. Individual collagen fibrils measured 20–30 nm in diameter and were interpreted as collagen type II. The border between the inner and intermediate zones was sometimes sharp and other times more difficult to distinguish because it contained a mixture of collagen types I and II (Fig. 5D).

**Figure 6:**
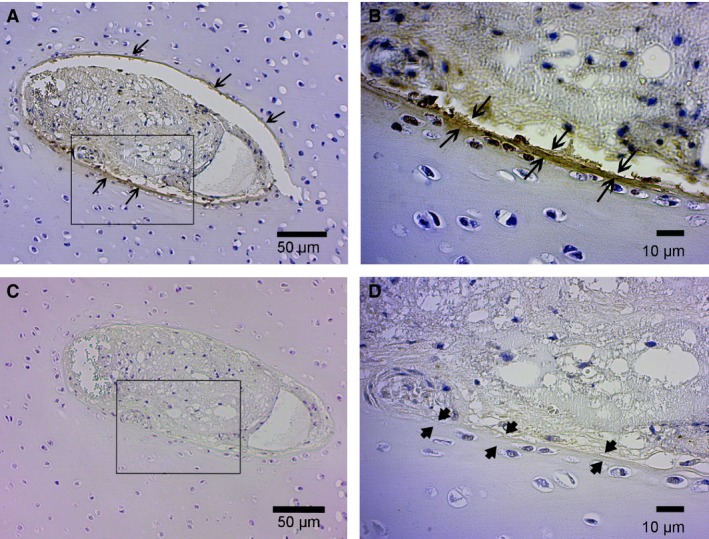
Immunostaining for type I collagen (A, B) and corresponding negative control (C, D). Distal femoral epiphysis, medial trochlear ridge. Thirty‐five‐day‐old Thoroughbred. Proliferative zone. Patent canal. (A) 100 ×. The image shows a cartilage canal surrounded by a narrow zone of matrix that stains immunopositive for collagen type I (brown colour; long arrows). The remaining cartilage matrix stains immunonegative for collagen type I. (B) 400 ×. Higher magnification of the same canals indicated by the black outline in (A). The image shows the innermost zone of matrix surrounding the cartilage canal staining immunopositive for collagen type I (brown colour; between long arrows). (C) 100 ×. The image shows the negative control of the same canal as in (A). (D) 400 ×. Higher magnification of the same canal indicated by the black outline in (C) showing the absence of brown stain (between the short arrows) in the same location that stained immunopositive for collagen type I in (B).

The eosinophilic ring corresponded to the fibrils that were interpreted as collagen type I with TEM. In immunostained sections, it was confirmed that some of the cartilage canals were surrounded by a thin zone of matrix that stained immunopositive for collagen type I compared with the immunonegative matrix surrounding other cartilage canals or between adjacent cartilage canals (Fig. 6A–D). Positive immunostaining for collagen type I was also observed in the matrix surrounding mesenchymal cells and in the walls of large arterioles inside cartilage canals. Collagen type I immunostaining was weak or absent in the area surrounding chondrifying canals.

## Discussion

To the authors' knowledge, the current study is the first ultrastructural description of epiphyseal cartilage canals in horses. The main finding was that there were two types of discontinuity in the endothelium of equine epiphyseal cartilage canal vessels: fenestrations were observed in a patent cartilage canal in the 118‐day‐old foal; and gaps were observed in chondrifying cartilage canals in all TEM‐examined foals.

### Discontinuities in the endothelium of patent cartilage canals

There are several possible explanations for the fact that fenestrations were observed in the 118‐day‐old foal and not in the other two foals where endothelium was examined by TEM. The cartilage canal where fenestrations were observed was located deep within the epiphyseal growth cartilage. This mirrors previous observations that fenestrations were more common deep compared with superficial in the growth cartilage of chicks (Hunt et al. 1979; Howlett, 1980). Deep canals may have failed to be included in the two foals where fenestrations were not observed because the cartilage was sampled superficial to the ossification front in order to avoid decalcification. Alternatively, fenestrations were observed in 4‐week‐old (Hunt et al. 1979) and 7‐week‐old (Howlett, 1980) chicks, but were not mentioned when chick embryos were examined with TEM (Blumer et al. 2004a). The 118‐day‐old foal was the oldest foal in which endothelium was examined with TEM and, together with the observations from chicks (Hunt et al. 1979; Howlett, 1980; Blumer et al. 2004a), this potentially suggests that fenestrations can be an age‐related phenomenon.

The current study included relatively few individuals, and it is not possible to conclude whether fenestrations are less common in the cartilage canal vessels of horses, compared with other species. In addition to being constitutively present in the vessels of some organs, fenestrations can represent a dynamic response to a wide variety of physiological, chemical and pathological stimuli, including cytokines and inflammatory mediators (Cogger et al. 2013). Vascular endothelial growth factor (VEGF) is considered a major cytokine in the regulation of fenestrations (Cogger et al. 2013), and VEGF expression is known to be high in the hypertrophic zone of growth cartilage (Carlevaro et al. 2000; Alvarez et al. 2005), i.e. the approximate depth level where fenestrations were currently observed. Experimental studies do not necessarily include non‐infected controls, and it can therefore be difficult to be sure of the extent to which endothelial discontinuities were present before bacteria were injected (Speers & Nade, 1985; Alderson et al. 1986). Bacteria in the circulation may induce cytokine release and inflammation, thus increasing the number of fenestrations (Cogger et al. 2013). A generalised increase in the number of fenestrations could explain the fact that lesions occurred in joints distant from the injected joint in experimentally infected pigs (Denecke & Trautwein, 1986; Denecke et al. 1986). Similarly, fragments were found in joints that were not diagnosed with septic arthritis in horses that survived infection before 6 months old (Hendrickson et al. [Link]). Indeed, while some bacteria are capable of triggering VEGF, bacteria in the circulation may also release factors that lead to formation of temporary channels, open up gaps between adjacent endothelial cells or trigger endothelial apoptosis or lysis (Edwards & Massey, 2011). It therefore seems that irrespective of the extent to which they were present before bacteraemia (Speers & Nade, 1985; Alderson et al. 1986), there are several reasons why the number of endothelial discontinuities may increase once bacteria have entered the circulation, thus exacerbating the risk of binding in bacteraemic compared with normal foals. The foal in which fenestrations were observed in the current study was killed due to complex clinical disease, including *Parascaris equorum* impaction and bladder rupture, and had signs of systemic inflammation. With the current methodology, it was not possible to answer whether the observed fenestrations represented constitutively present (Cogger et al. 2013) or disease‐induced fenestrations (Edwards & Massey, 2011). Fenestrations were observed in equine epiphyseal cartilage canal vessels, and bacterial binding can therefore probably occur in horses by mechanisms that are similar to those previously demonstrated in pigs and chickens (Emslie & Nade, 1983; Speers & Nade, 1985; Alderson et al. 1986; Denecke & Trautwein, 1986; Denecke et al. 1986). Bacterial binding is likely to have the same consequence in horses as in pigs and chickens, i.e. occlusion of the vessel (Emslie & Nade, 1983; Speers & Nade, 1985; Alderson et al. 1986; Denecke & Trautwein, 1986; Denecke et al. 1986). Vascular failure has been demonstrated to lead to ischaemic chondronecrosis, OC and OCD in foals (Olstad et al. 2013), and bacterial binding followed by occlusion and ischaemia provides a potential explanation for the OCD‐like flaps and fragments that were observed in the clinical reports of cases with septic arthritis (Hance et al. 1993; Haggett et al. 2012).

### Discontinuities in the endothelium of chondrifying cartilage canals

In the current study, an additional type of discontinuity was also observed in terms of endothelial cells detaching from the vascular basement membrane leading to gap formation in the vessels of early chondrifying cartilage canals. Ultrastructural studies have tended to focus on patent, rather than chondrifying, canals. Two types of discontinuity were previously observed in sheep foetuses: 60‐nm‐diameter pores and wider discontinuities; the latter tended to be observed superficially within the cartilage and as this is where chondrification begins, it potentially agrees with the currently observed gaps in chondrifying canals (Stockwell, 1971). Degenerative changes including contraction of endothelial cells and disruption of the vascular basement membrane were also observed when chondrifying cartilage canals were described in a study of 25‐ and 45‐kg bodyweight pigs (Woodard et al. 1987). As discussed above, different kinds of discontinuities may occur, and enable direct contact and binding between bacterial surface proteins and components of the ECM of growth cartilage. One might think that occlusion of a vessel in a chondrifying cartilage canal will not result in ischaemic chondronecrosis, because chondrification implies that the blood supply is no longer needed (Haines, 1974; Carlson et al. 1991, 1995). However, chondrification starts superficially and proceeds in a proximal direction along the cartilage canal (Ytrehus et al. 2004a; Olstad et al. 2007), and the afferent arteriole and efferent venule enter and exit the epiphyseal growth cartilage through the same canal (Wilsman & Van Sickle, 1972; Hayashi, 1992). Occlusion of a venule superficially within the chondrifying canal may therefore result in retrograde stasis and failure of the afferent arteriole at a depth where the chondrocytes are still dependent on a blood supply and susceptible to ischaemic chondronecrosis (Carlson et al. 1991; Olstad et al. 2013). In the study of horses that survived infection before 6 months old, it was significantly more common for horses hospitalised for infection ≤ 30 days old to have osteochondral fragments in the fetlock compared with the hock joint (Hendrickson et al. [Link]). The principal difference between these two joints is that a greater proportion of the cartilage canals undergo chondrification in the window from birth to 35 days old in the fetlock (Olstad et al. 2009) compared with the hock joint (Olstad et al. 2008b). It is therefore theoretically possible that the higher prevalence of fragments in the fetlock compared with the hock joint of infection survivors (Hendrickson et al. [Link]) reflects bacterial binding and vascular occlusion via discontinuities in chondrifying, rather than in patent, cartilage canal vessels.

### Cellular wall

Some of the current observed cartilage canals were surrounded by a hypercellular rim. An apparently similar hypercellular rim was previously observed in several different studies (Lutfi, 1970a; Stockwell, 1971; Wilsman & Van Sickle, 1972; Haines, 1974; Doménech‐Ratto et al. 1999). In one of the LM studies, the presence of increased numbers of flattened cells around cartilage canals was understandably described as the cartilage canal having a discontinuous cellular wall (Doménech‐Ratto et al. 1999). However, observations made in the current and previous TEM studies (Lutfi, 1970a; Wilsman & Van Sickle, 1972; Haines, 1974) indicate that the hypercellular rim represents differentiation of perivascular mesenchymal cells into chondrocytes that contribute to growth of the cartilage model. These morphological observations are supported by an experimental study where the distribution of serially administered tritiated thymidine, which labels dividing cells, indicated that cells immediately adjacent to cartilage canals were the result of division of perivascular mesenchymal cells within the cartilage canal (Lutfi, 1970b). In the current study, the hypercellular rim was present inconsistently and predominantly in the youngest foal. Cells within the rim were observed to produce ECM molecules similar to those produced by chondrocytes in the remainder of the epiphyseal growth cartilage. There is therefore no reason to believe that the risk of bacterial binding and vascular occlusion is different between cartilage canals with a hypercellular rim compared with cartilage canals without.

### Acellular collagen type I wall and bacterial binding

In the currently studied foals, the majority of cartilage canals were surrounded by an eosinophilic ring that was immunohistochemically confirmed to consist of collagen type I. This agrees with previous observations from LM studies in foals (Olstad et al. 2007) and chickens (Lutfi, 1970a), and TEM studies in humans (Haines, 1974), chickens (Lutfi, 1970a; Doménech‐Ratto et al. 1999) and sheep (Stockwell, 1971). A continuous layer of collagen I was found immediately surrounding cartilage canals in mice (Blumer et al. 2007) and chickens (Blumer et al. 2004a,b), and appeared thicker and more obvious around deep compared with superficial cartilage canals (Blumer et al. 2006). Bacterial species express surface proteins with binding affinity for specific molecules (Chagnot et al. 2012), including different components of the ECM of epiphyseal growth cartilage, such as collagen, fibronectin (Ekman & Heinegard, 1992) and laminin (Ganey et al. 1995). The range of surface proteins varies between different bacterial species, and the species that express surface proteins with a binding affinity for collagen type II are not necessarily the same species as those that express proteins with a binding affinity for collagen type I (Chagnot et al. 2012). As cartilage canals were surrounded by collagen type I in addition to collagen type II, this potentially means that discontinuities render vessels susceptible to binding and occlusion by bacterial species with a binding affinity for collagen type I, in addition to the species with a binding affinity for collagen type II.

### Acellular collagen type I wall and OC

The current study is the first where the fact that individual cartilage canals within the same histological section can be surrounded by different types of collagen has been quantified in the horse (Table 3). Historically, it was suggested that primary disease of collagen led to structurally weakened cartilage and OCD in horses (Semevolos et al. 2001; Laverty et al. 2002; van de Lest et al. 2004). It was, however, difficult to explain why lesions should occur multi‐focally at predilection sites (McIlwraith, 1993), and once it was demonstrated that vascular failure led to OCD (Olstad et al. 2013), focus shifted to ask whether primary disease of collagen was capable of causing vascular failure (Laverty & Girard, 2013). Collagen structure was observed to be different in the area immediately around the cartilage canals compared with the area between cartilage canals (Henson et al. 1996; Lecocq et al. 2008). In spontaneously occurring vascular failure, vessels in a single cartilage canal fail at the same time as vessels in adjacent cartilage canals at the same stage of development remain intact (Olstad et al. 2008a,b). An explanation for why vascular failure affects some cartilage canals and not others is therefore still needed, and it is considered that the results of the current study provide the first real opportunity to generate working hypotheses for how this might occur. Initially, the fact that it is vessels within a minority of cartilage canals that fail (Olstad et al. 2008a,b) combined with the observation that the majority of cartilage canals were surrounded by collagen type I prompts the suggestion that being surrounded by collagen type II may render a cartilage canal more susceptible to failure. This and other hypotheses are currently the subject of further research in the authors' labs.

## Conclusion

Two types of discontinuity were observed in the endothelium of equine epiphyseal cartilage canal vessels: fenestrations were observed in a patent cartilage canal in the 118‐day‐old foal; and gaps were observed in chondrifying cartilage canals in all TEM‐examined foals. Canals were not surrounded by any cellular wall, but a large proportion was surrounded by an acellular wall consisting of collagen type I. Bacterial binding can therefore probably occur in horses by mechanisms that are similar to those previously demonstrated in pigs and chickens.

## Author contributions

I.R.H. contributed to the study design, study execution, data analysis and interpretation and writing of the manuscript. K.O. and S.E. contributed to the study design, study execution, data analysis and interpretation. K. H. performed the data preparation and contributed to the data interpretation for TEM. N.I.D. contributed to the study design and data interpretation. All authors contributed to the preparation of the manuscript, critically revised the manuscript and have approved the final version of the manuscript.

## Conflict of interests

The authors declare that they have no competing interests.

## Supporting information


**Data S1**. Immunohistochemistry.
