# Supplementary material for: Discontinuities in the endothelium of epiphyseal cartilage canals and relevance to joint disease in foals
Source: J Anat. 2015 Oct 15;228(1):162–75. doi: 10.1111/joa.12391 (PMC4694163; doi:10.1111/joa.12391)
Supplement: Supplementary file 1 — Data S1. Immunohistochemistry. [file JOA-228-162-s001.docx]

**Supplemental Methods**

**Immunohistochemistry**

Specimens were sectioned for immuno-localization of type I collagen and mounted onto slides, deparaffinised, rehydrated in descending grades of ethanol and washed in phosphate-buffered saline (PBS; 0.01 M, pH 7.4. Medicago AB, Uppsala, Sweden). PBS was used for all dilutions below unless stated otherwise. Endogenous peroxidase activity was quenched by incubating the sections for 5 minutes in 3 % hydrogen peroxide in PBS. Following washing in PBS, non-specific binding was blocked by incubating the sections in 2% normal goat serum (DAKO, X0907) in PBS for 30 minutes at room temperature (RT). The sections were then dried, and incubated with a rabbit polyclonal antiserum against equine collagen type I (Abcam, ab34710) added at a concentration of 10µg/ml for 60 minutes RT. After rinsing in PBS, the horseradish peroxidase (HRP)-conjugated secondary antibodies directed against rabbit and mouse immunoglobulins (Dako Real EnVision™ Detection System, k5007, ready-to-use kit) were added and the sections incubated at 30 minutes RT. Sections were then rinsed in PBS. Visualisation was performed by adding the colour developer 3,3-diaminobenzidine (DAB+ Cromogen Dako EnVision) for 5-10 minutes RT. Slides were counterstained with haematoxylin. Negative (isotope) controls were performed by substituting the primary antibody with nonimmune-rabbit-serum (Rabbit Immunoglobulin Fraction X0936, DAKO) using the same concentration as for the primary antibody. Positive controls were osteoid from the secondary ossification centre of the epiphysis of the 35 days old foal.

The sections were evaluated subjectively using Nikon Eclipse E600 microscope and NIS Elements Basic Research software version 3.22.11 (Nikon Instruments Inc, Melville, NY, USA).
